# Relationships between Community Level Functional Traits of Trees and Seedlings during Secondary Succession in a Tropical Lowland Rainforest

**DOI:** 10.1371/journal.pone.0132849

**Published:** 2015-07-14

**Authors:** XingHui Lu, RunGuo Zang, JiHong Huang

**Affiliations:** 1 Key Laboratory of Forest Ecology and Environment of State Forestry Administration, Institute of Forest Ecology, Environment and Protection, Chinese Academy of Forestry, Beijing, China; 2 Co-Innovation Center for Sustainable Forestry in Southern China, Nanjing Forestry University, Nanjing, Jiangsu, China; Shandong University, CHINA

## Abstract

Most of the previous studies on functional traits focus exclusively on either seedlings or trees. Little knowledge exists on the relationships between community level functional traits of trees and seedlings during succession. Here, we examine variations of the community-level functional traits for trees and seedlings and their correlations along a secondary successional and environmental gradient in a tropical lowland rainforest after shifting cultivation. The results showed that the dynamic patterns in community level functional traits of seedlings were generally consistent with those of the trees during secondary succession. Compared with seedlings, community level traits for trees were less affected by abiotic factors during secondary succession. Correlations between community level functional traits of trees and seedlings were significant for: leaf dry matter content and leaf nitrogen concentration in the 18-year-old fallow; leaf chlorophyll content in the 30-year-old fallow; specific leaf area, leaf dry matter content and leaf nitrogen concentration in the 60-year-old fallow; and leaf nitrogen concentration in old growth. However, these traits except specific leaf area for the tree and seedling communities were all significantly correlated if all the successional stages were combined. Our results suggest that the correlations between community level functional traits of trees and those of seedlings depend on the actual traits and the successional stages examined. However, if all the four successional stages are combined, then four out of five of the community level functional traits for trees could be well predicted by those of the seedlings in the tropical lowland rain forest.

## Introduction

Plant functional traits are indicators of their effects on processes contributing to ecosystem functioning. Functional trait-based approaches have proven to be an effective tool to understand the vegetation dynamics patterns and ecosystem processes, and have been widely used in community ecology [1–3]. Trait-based approaches are now used in studies ranging from the level of organisms to that of ecosystems. Until recently, many studies on plant functional traits have focused on differences in mean values of traits between- and within-species at the individual species level [4,5]. Indeed, community-weighted means of trait values (CWM) largely determine how individual plant species contribute to ecosystem processes at the community level [6]. This supports the “mass ration hypothesis”, which proposes that ecosystem processes are largely determined by the dominant species functional traits in a community [6].

As plants develop, they increase in both size and functions [7]. There is ample evidence of changes in functional traits from the seedling to the tree phase of woody plants. For example, leaf structural traits, nutrient content, and palatability varied significantly from seedling to tree [7–9]. Specific leaf area (SLA) decreased significantly from seedlings that experience low light environment to trees that acclimated to high light intensities in the upper canopy [8]. Leaf traits are positively related to seedling potential growth across species [10] but are weakly or not related to the growth of adult trees [11]. However, studies have shown that some functional traits of trees and seedlings are strongly related, as well as their variation patterns during succession [12,13]. SLA of tree decreased with the successional stage [14]. Similar patterns have been observed for seedlings in a tropical lowland rainforest [15]. These previous studies suggest that even though there are differences in functional traits between trees and seedlings, the variation patterns during succession are consistent.

Most of the previous findings on plant functional traits generally focus on one particular stage (seedlings or trees). However, many traits (such as root/shoot ratio and the traits related with biomass allocation), while easy for the seedlings, are difficult to measure for trees in forest ecosystems. There is no literature on whether community level functional traits of trees be predicted by those of seedlings. If this were possible, then we could sample functional traits more easily for seedlings instead of trees in our explorations of community level functional traits of trees in forest ecosystems. Here, we examine variations of the community-level functional traits for trees and seedlings and their correlations along a secondary successional gradient in a tropical lowland rainforest after shifting cultivation. In this study, we seek to answer three questions: 1) how do functional traits of tree (diameter at breast height (DBH) ≥ 1 cm) and seedling (DBH < 1 cm) communities vary during secondary succession; 2) how are functional traits of tree and seedling communities affected by environment; 3) can functional traits of the tree community be predicted by those of the seedling community.

## Materials and Methods

### Study site

This study was conducted in Bawangling forest region (18°52′–19°12′ N, 108°53′–109°20′ E) on Hainan Island, south China, located at the northern edge of Asian tropical forest. The Administration Bureau of Hainan Bawangling National Natural Reserve gave permission to conduct the study in this area. The Bawangling forest is *ca* 500 km^2^, with an elevation range of *ca* 100–1654 m above sea level (asl). The vegetation in the region varies with elevation. This study was conducted in the tropical lowland rain forest (< 800 m asl). The mean annual temperature is 23.6°C, and annual precipitation is 1677 mm with a marked wet season from May to October and a dry season from November to April. The old growth forests are dominated by the canopy tree species *Vatica mangachapoi* Blanco (Dipterocarpaceae), *Litchi chinensis* Sonnerat (Sapindaceae), and *Homalium ceylanicum* (Gardner) Bentham (Salicaceae), with a diverse array of co-occurring species. The number of species and individuals in old growth forest plot were 184.78 ± 49.44 and 52.34 ± 7.50. The mean DBH and height of trees in old growth forest plot were 6.31 ± 1.26 m and 5.44 ± 0.6 m.

The field investigation was conducted in the tropical lowland rainforest that had naturally recovered after shifting cultivation in four stages of succession (18-year-old fallow, 30-year-old fallow, 60-year-old fallow, and old-growth forest). The information about these fallows is listed in S1 Table. 50 plots (20 m × 20 m) were sampled randomly in every stage of succession. The plots were more than 20 m apart from each other. Information on the history of land use of the plots was obtained from management records of Bawangling Bureau of Forestry of Hainan. These plots have similar topographic conditions. They are located in low hills with slopes of about 15 degrees, and their elevations range from 412 to 626 m.

All woody plants with DBH ≥ 1 cm in each plot were identified and measured. At the center of each plot, we established a 2 × 2 m seedling plot within which we tagged, measured and identified all woody seedlings < 1 cm DBH. All tree and seedling species were sampled with 10 individuals to measure functional traits. The information about the tree and seedling community in each stage is listed in S2 Table. This study did not involve endangered or protected species.

The sampling areas for seedlings and trees are different in forest ecosystems, especially in tropical forests. There are too many seedling individuals in a 1-ha tropical rainforest plot and it is almost impossible to complete sampling for all these seedlings. Therefore, the area of seedling plots in tropical forest research is always set to 1 × 1 m or 2 × 2 m [16–19]. This sampling area encompasses the density and richness of seedlings. In the great majority of studies, sampling areas of trees and seedlings are usually different in investigating community structure. For instance, Norden et al. [20] identified trees in 100 10 × 10 m quadrats (1 ha), saplings in 200 5 × 5 m quadrats (0.5 ha), and seedlings in 1000 2× 1 m quadrats (0.2 ha). Meanwhile, Gonzalez et al. [21] measured seedlings in 4 × 4 m plot, and trees in 50 ×50 m plots to study the shifts in species and phylogenetic diversity between seedling and tree communities. The smaller sampling scale for seedlings than for trees is well documented for exploring trees and seedlings in the same tropical forest ecosystem.

For each individual, two recently-expanded leaves, including petioles and rachises of compound leaves were collected. Leaf surface area was measured with a leaf area meter (LI-COR 3100C Area Meter, LI-COR, USA). Leaf dry mass was measured by drying to constant mass at 60°C (around 72 h), and SLA (cm^2^ g^-1^) was calculated for each leaf as the ratio of leaf surface area to leaf mass. Leaf dry matter content (LDMC, g g^-1^) was the oven-dry mass divided by its fresh mass. Leaf chlorophyll content (CC, SPAD) was estimated using three values per lamina from a SPAD 503 Plus meter (Konica Minolta, Osaka, Japan). Leaf nutrition characteristics, including leaf nitrogen concentration per mass (LNC, mg g^-1^) and leaf phosphorus concentration per mass (LPC, mg g^-1^), were measured by micro-Kjeldahl digestion method [22].

### Environmental factors

At the center of each plot, hemispherical canopy photographs were taken at 1 m above ground level using a fish-eye lens (HMV1v8, Delta-T Devices Ltd., Cambridge, UK). Canopy cover was measured from each photograph as the percentage of closed-canopy pixels using the Gap Light Analyzer software. Canopy Openness (CO, %) was 1− canopy cover.

We collected two soil samples with 0–20 cm depth at the center of each plot to measure soil characters. One fresh soil sample was weighted immediately after sample, dried at 105°C to a constant weight, and weighed again to determine soil water content. The other soil sample was used for nutrient analysis, dried at ambient temperature in the lab to prevent the volatilization of nitrogen compounds that can occur when samples are dried at high temperature. Measurements included soil pH, soil organic matter content (SOM; g kg ^-1^), total nitrogen content (TN; g kg ^-1^), total phosphorus content (TP; g kg ^-1^), available nitrogen content (AN; mg kg ^-1^), available phosphorus content (AP; mg kg ^-1^).

### Statistical analyses

We calculated the CWM of seedling communities on the basis of two databases: seedling-trait database and plot-seedling abundance database. We used the two databases to construct a plot-trait database to calculate the CWM of seedling communities. Using the same method, we calculated the CWM of tree communities on the basis of two databases: tree-trait database and plot-tree abundance database. The CWM value, which is proportional to species abundance, is more appropriate than the unweight mean in characterizing trait variations at the community level. These calculations were based on the Fd_var_ index using the FD package in R with the function dbFD. It is calculated as follows:

$$\text{CWM}=\sum_{i=1}^{s}W_{i}\times X_{i}$$ (1)

where *S* is the total number of species, *W*_*i*_ is the relative abundance of the i^th^ species and *X*_*i*_ is the trait value of the i^th^ species. Relative abundance was measured as the ratio of individual numbers to total individual numbers in a plot.

To allow for parametric statistical analyses, we log-transformed traits values before analysis. We tested for the effects of succession and life phase (tree or seedling) on traits with analysis of covariance, with traits succession ×life phase as an explanatory factor. Differences in environmental factors among the four successional stages were assessed using one-way analysis of variance. If variation was significant (*P* < 0.05), we performed Tukey honest significant difference multiple comparisons to determine the significance of differences among stages.

We used fourth corner analysis to test the significance of the link between species distribution, traits and environmental factors. This analysis tests for correlations between traits and environment by linking a site × environment matrix (R) via a site × species abundance matrix (L) to a trait × species matrix (Q). We ran the fourth corner analysis using a model 4 permutation to test the null hypothesis that the species composition of samples with fixed environmental conditions is not influenced by the species characteristics [23].

Linear regression analyses were performed in order to quantify the relationships between community level functional traits of trees and seedlings during succession.

We have used ANOSIM (analysis of similarities) to test whether species composition differed between the tree and the seedling community for each successional stage using the anosim function in the “vegan” package in R. similarity of species composition between the tree and seedling community. Multivariate stepwise regression was applied to test the relationship between environmental factor and the range of community level functional traits (trees’ minus seedlings’).

## Results

### Variation patterns of community level functional traits during succession

**Fig 1:**
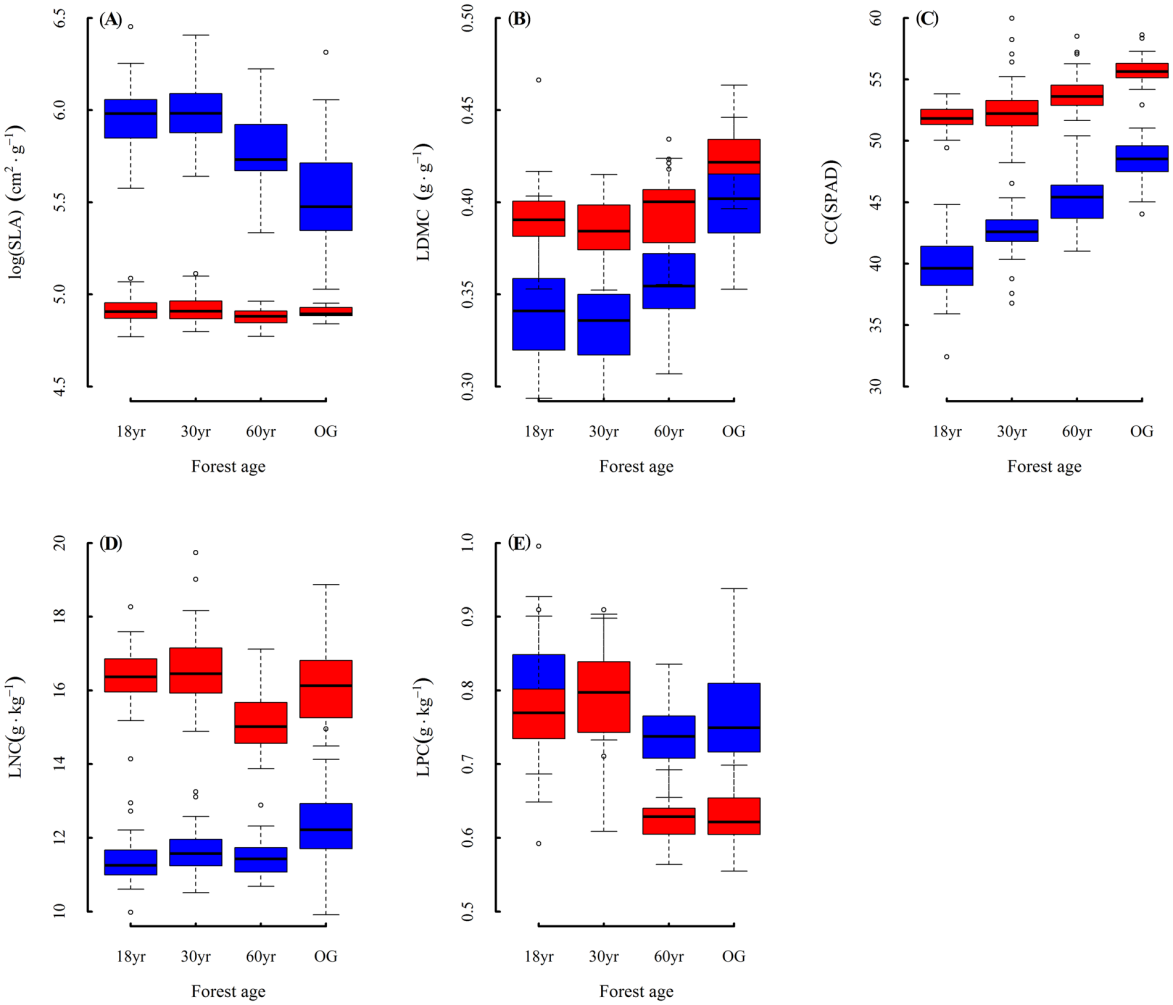
Variation of functional traits between tree (red box) and seedling (blue box) community during succession. (A) Speciefic leaf area; (B) leaf dry matter content; (C) leaf total chlorophyll content; (D) leaf nitrogen concentration per mass; (E) leaf phosphorus concentration per mass.18yr, 18-year-old fallow; 30yr, 30-year-old fallow; 60yr, 60-year-old fallow; OG, old growth forest.

The variation patterns of functional traits for tree and seedling communities during succession were similar (Fig 1). Tree community SLA was lowest in the 60-year-old fallow, while seedling community SLA decreased during succession. Leaf dry matter content of both tree and seedling communities were lowest in 30-year-old fallow, and then increased with succession. Leaf total chlorophyll content increased with succession in both tree and seedling communities. Leaf nitrogen concentration and leaf phosphorus concentration were lowest in the 60-year-old fallow both in tree and seedling communities.

There were significant differences in all functional traits between tree and seedling communities (Fig 1). The SLA of seedling communities was significantly lower than those of the tree communities in all successional stages. In contrast, tree communities had significantly higher CC and LNC in all successional stages. The LDMC of seedling communities were generally lower than the tree communities, except in old growth forest. Seedling communities had generally higher LPC than tree communities, except in 30-year-old fallow.

### Variation of environmental factors during succession

Most environmental factors changed significantly with successional stages. Canopy openness decreased during succession (Table 1). Old growth forest had the lowest canopy openness, whereas 18-year-old fallow had the highest value. 18- and 30-year-old fallows had lower soil water content, and the later succession (60-year-old fallow and old growth forest) fallows had higher values. Total nitrogen content, available nitrogen content, available phosphorus content, and soil organic matter content were highest in 30-year-old fallow, and were lowest in old growth forest. Total phosphorus content decreased during succession. Soil pH was highest in 30- and 60-year-old fallow and lowest in 18-year-old fallow and old growth forest.

**Table 1:** Variations of environmental factors during succession.

	18-yr	30-yr	60-yr	Old growth
**CO (%)**	39.46 ± 10.80 a	24.62 ± 7.94 b	8.54 ± 2.99 c	6.71 ± 1.85c
**SWC (g g** ^**-1**^ **)**	12.50 ± 5.02 a	11.88 ± 3.59 a	13.51 ± 4.06 a	13.35 ± 2.41 a
**TN (g kg** ^**-1**^ **)**	1.48 ± 0.36 a	1.86 ± 0.41 b	1.38 ± 0.34 a	1.07 ± 0.18 c
**TP (g kg** ^**-1**^ **)**	0.37 ± 0.22 a	0.31 ± 0.06 a	0.30 ± 0.13 a	0.20 ± 0.09 b
**AN (mg kg** ^**-1**^ **)**	88.89 ± 22.94 a	98.25 ± 23.20 ab	85.86 ± 25.57 c	76.04 ± 24.95 bc
**AP (mg kg** ^**-1**^ **)**	9.75 ± 4.16 a	9.46 ± 5.42 a	6.86 ± 3.32 b	4.27 ± 1.73 c
**pH**	4.73 ± 0.17 a	4.90 ± 0.22 b	4.84 ± 0.15 b	4.74 ± 0.19 a
**SOM (g kg** ^**-1**^ **)**	45.22 ± 7.81 a	47.35 ± 6.39 a	39.12 ± 8.38 b	31.87 ± 5.63 c

The different letter (a, b, c) indicates significant differences (*P* < 0.05). Abbreviations of environmental factors: CO, canopy openness; SWC, soil water content; TN, total nitrogen content; TP, total phosphorus content; AN, available nitrogen content; AP, available phosphorus content; SOM, soil organic matter content. Abbreviations of forest age are given in Fig 1.

### Relationships between traits and environmental factors during succession

**Fig 2:**
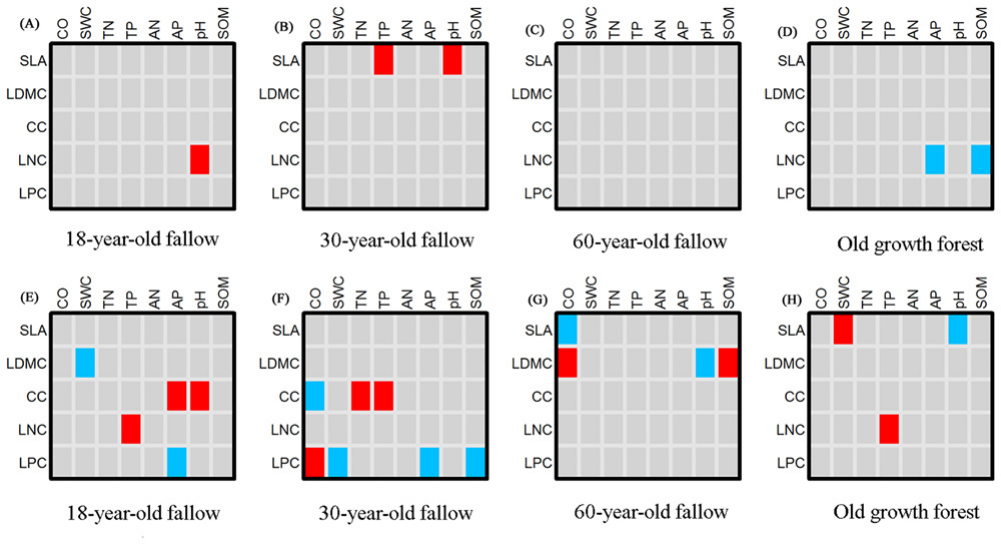
Output of the fourth corner analysis on the relationships between traits and abiotic factors during the secondary succession. (A) tree community in 18-year-old fallow; (B) tree community in 30-year-old fallow; (C) tree community in 60-year-old fallow; (D) tree community in old growth forest; (E) seedling community in 18-year-old fallow; (F) seedling community in 30-year-old fallow; (G) seedling community in 60-year-old fallow; (H) seedling community in old growth forest. Red fields represent significant positive relationships, blue fields represent significant negative relationships and gray fields represent non-significant relationships. Abbreviations of functional traits and environmental factors are given in Fig 1 and Table 1.

The fourth corner analysis showed the relationships between traits and environmental factors of tree and seedling communities during succession. Compared with seedling communities, tree community traits were more stable, and were less affected by environmental factors (Fig 2). Only two traits (LNC and SLA) of tree communities were significantly affected by environment during the four successional stages. Tree community LNC was positively related to soil pH in the 18-year-old fallow, whereas it was negatively related to AP and SOM in the old growth forests. Tree community SLA was positively related to TP and pH only in the 30-year-old fallow. In contrast, all traits of seedling community were generally significantly associated with environmental factors, although traits were affected by different factors in different successional stages. Seedling community SLA was significantly affected by the environment in later successional stages (60-year-old fallow and old growth forest). In 18-year-old fallow and old growth forest, LDMC and LNC were affected by the environment. In the earlier two stages (18- and 30-year-old fallow), CC and LPC were significantly associated with the environmental factors.

### Relationships between community level functional traits of trees and seedlings during succession

**Fig 3:**
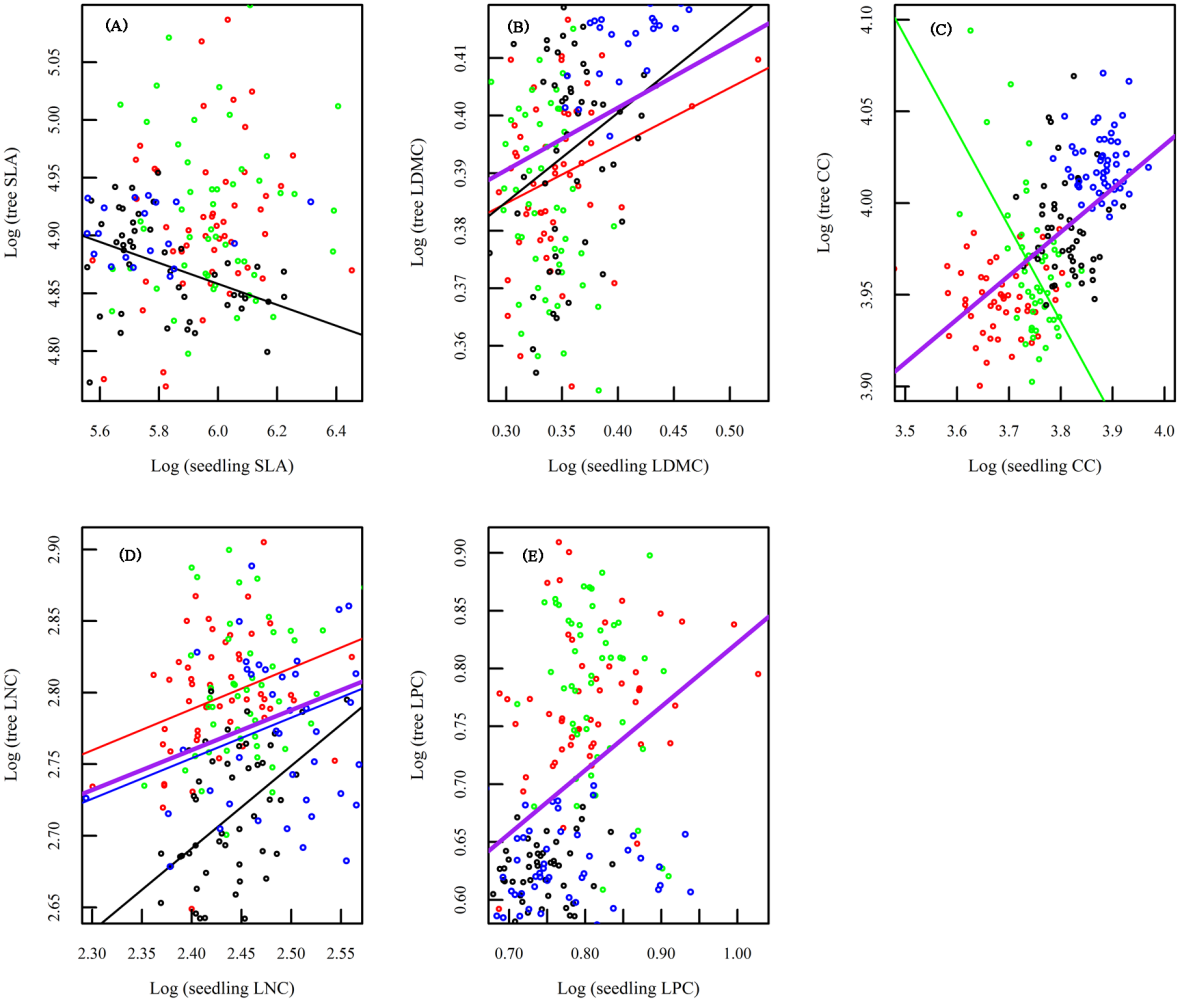
Relationships between functional traits of tree and seedling communities during the secondary succession. (A) specific leaf area of tree and seedling communities; (B) leaf dry matter content of tree and seedling communities; (C) leaf total chlorophyll content of tree and seedling communities; (D) leaf nitrogen concentration per mass of tree and seedling communities; (E) leaf phosphorus concentration per mass of tree and seedling communities. We only show the significant regression line. Red points and line represent 18-year-old fallow. Green points and line represent 30-year-old fallow. Black points and line represent 60-year-old fallow. Blue points and line represent old growth forest. Purple line represents total (all the four successional stages combined). * *P* < 0.05.

The results indicated that correlations between community level functional traits of trees and seedlings were significant for: LDMC and LNC in the 18-year-old fallow, CC in the 30-year-old fallow, SLA, LDMC and LNC in the 60-year-old fallow, and LNC in the old growth forest. However, with the exception of SLA, they were all significantly correlated if all the four successional stages were combined. Tree community SLA was negatively related to that of the seedling community in the 60-year-old fallow (Fig 3A, Table 2). Tree community LDMC was positively related to that of the seedling community in the 18- and 60-year-old fallows (Fig 3B, Table 2). Tree community CC was negatively related to that of the seedling community in the 30-year-old fallow (Fig 3C, Table 2). Tree community LNC was positively related to that of the seedling community in the 18- and 60-year-old fallows, as well as old growth forest (Fig 3D, Table 2). Tree community LPC was not significantly related to that of the seedling community during secondary succession (Fig 3E, Table 2). Most of the community level functional traits of trees can be predicted well by those of the seedlings if all the four successional stages are combined. With the exception of SLA, all four traits were significantly correlated between tree and seedling communities.

**Table 2:** Linear regressions (y = ax+b) between functional traits of tree communities (y) and those of the seedling communities (x) during the secondary succession.

	SLA	LDMC	CC	LNC	LPC
**18yr seedling -18yr tree community**	*P* = 0.17	**y = 0.10x+0.35** *	*P* = 0.53	**y = 0.28x+2.09** *	*P* = 0.07
**30yr seedling-30yr tree community**	*P* = 0.89	*P* = 0.08	**y = -0.51x+5.90** *	*P* = 0.79	*P* = 0.12
**60yr seedling-60yr tree community**	**y = -0.09x+5.40** *	**y = 0.15x+0.33** *	*P* = 0.86	**y = 0.57x+1.30** *	*P* = 0.09
**OG seedling-OGtree community**	*P* = 0.92	*P* = 0.9	*P* = 0.69	**y = 0.28x+2.07** *	*P* = 0.47
**Total seedling-total tree community**	*P* = 0.39	**y = 0.11x+0.36** *	**y = 0.24x+3.08** *	**y = 0.28x+2.09** *	**y = 0.55x+0.27** *

Abbreviations of functional traits are given in Fig 1.

* *P*< 0.05.

## Discussion

### Patterns of changes in traits during secondary succession

Species that occupy different positions along succession often differ markedly in their functional traits. Ellsworth and Reich [24] found that photosynthesis and leaf nitrogen decreased during secondary forest succession owing to the combination of changes in resource availability and species composition. SLA of trees decreased with the successional position in a tropical lowland moist forest in the Bolivian Amazon [20]. Similar patterns have been observed for seedlings in a tropical lowland rainforest in China [21]. In the present study, we found that community level functional traits of trees and seedlings had similar variation patterns during succession (Fig 1).

A higher SLA is important for both tree and seedling growing in resource-rich environments in early succession [25]. High SLA has been associated with short leaf turnover time and high relative growth rate, which may permit rapid growth rate of early successional trees and seedlings. Larger plants with larger leaves require greater support, causing ontogenetic declines in SLA [13]. Leaves of trees and seedlings with high LDMC in late succession tend to be relatively tough, which could be advantageous in resistance to physical hazards (e.g. herbivory, wind, hail) [26]. Trees and seedlings in late succession had higher CC to increase the capacity for light absorption. Similarity, Rijkers et al. [27] found that species grown in a shade environment have higher chlorophyll content than species grown under sun light. Leaf N and P concentrations are closely correlated with photosynthetic rates per mass [24,28,29]. High LNC and LPC allow early successional tree and seedling to gain high photosynthetic rates.

### Differing responses of seedlings and trees to environmental conditions during secondary succession

Poorter and Markesteijn [30] have compared functional traits of seedlings grown in dry and moist tropical forest. The results showed that seedlings invest biomass to acquire limiting resources; for example, drier forest seedlings invest more biomass into roots to enhance water capture, and moist forest seedlings invest more biomass into leaves and stems to acquire more light. This study proves the important role of environmental filter in shaping seedling functional traits. Environmental conditions experienced by trees are often very different from those experienced by seedlings [31]. The main threats to survival may change during ontogeny. Seedlings must maintain a positive carbon balance to survive and growth in the shade environment, but increasing size may engender greater resilience to accidental damage, attack by herbivores and drought [32]. The different environments of tree and seedling may play a different role in shaping functional traits.

We speculate that dominant environmental factors were different between trees and seedlings. For example, light is likely to be higher for trees than for seedlings, and wind exposure and physical abrasion are likely to be higher in canopy environments than in the understory [7]. Our fourth corner analysis results showed that relationships of traits and environmental factors were different between trees and seedlings. None of the tree traits were associated with CO, whereas four traits (SLA, LDMC, CC and LPC) of seedlings were associated with CO during the four successional stages. These results were due to the fact that light is a limiting factors for seedlings but not for trees. Only two traits (LNC and SLA) of trees were affected by environment during the four successional stages. Soil pH, phosphorus content, and SOM are important determinants of functional traits of trees. Although the relationships between traits and environment of seedlings were not the same in different successional stages, the relationships were more closely aligned with trees.

We hypothesized that the relationships between functional traits and environmental factors would disappear during the seedling-to-tree transition owing to the decreasing role of the environmental factors. The relationships of traits to the environmental factors in tree and seedling communities suggest that environmental filtering is an important mechanism shaping the seedling community's functional traits, although less important for tree community [33]. The functional traits of the tree community might be susceptible to other factors, such as biotic competition, rather than the abiotic environment.

The results showed that there were significant differences in species composition for tree and seedling communities in each successional stage (ANOSIM test, P < 0.05 in all cases). The similarity indexes of species composition between the tree and seedling communities were low (S1 Fig). So our tests from the two approaches suggest that the correlations of functional traits between seedling and tree communities were not due to the taxonomic correspondences between seedlings and trees. S3 Table suggested that the differences of tree and seedling functional traits at community level were not due to the environmental factors. In conclusion, most of the functional traits of the forest as a whole could be well represented by those of the seedling community. Since both the traits and the environmental / resources conditions change during succession, the responses of seedlings and trees to these changes might be different or similar. The changes in resource availability influence seedling traits, and the traits might be limited by the changed resources during a seedling community becomes a tree community. What we mainly concern is the variations of functional traits at community level, which are not only related with the abiotic environments and also related with the biotic interactions as the succession, proceed. Our study just presented the relationships between community level functional traits of trees and seedlings during secondary succession. As for the actual mechanisms of the relationships, we need to do more work on the ecophysiology of the component species, species interactions, ontogenetic shifts, and phylogenetic relations ect.

### Can the community level functional traits of trees be predicted by those of seedlings?

Cornelissen et al. [12] have found that predicting traits of field trees from laboratory seedlings is possible to some extent in both British and Spanish woody flora. The similar results also had been presented for red oak species (*Quercusrubra*) at the Harvard Forest in Petersham, Massachusetts [9]. These previous studies on predicting traits of trees from seedlings were generally conducted at the species level. In our study, we analyzed the correlations of functional traits between tree and seedling communities. If the community level functional traits of trees can be predicted by those of seedlings, we can do the sampling of functional traits more easily for seedlings and then use the seedling data to explore community level functional traits of trees in forest ecosystems.

Our results suggest that we cannot rule out the possibility that predicting functional traits of tree community from seedling community. However, the evidence presented here suggests that the prediction of functional traits from seedling communities to tree communities depends on the specific traits and the successional stages in the lowland rain forest. For instance, SLA of tree communities had significant correlation with that of seedling communities only in the 60-year-old fallow. However, LNC of tree community was positively related to that of the seedling community in 18- and 60-year-old fallows, as well as old growth forest. If we combined the four successional stages, most of community level functional traits of trees can be predicted well by those of seedlings. Our results enhance our understanding of the functional relationships between tree and the seedling communities during succession in forest ecosystems. In addition, our results provide good evidence that the community level functional traits of trees can be predicted by those of the seedlings, at least for some of the traits in some successional stages of forest development. This should provide guidance for sampling designs for measurement of functional traits and insights into the understanding of predicting properties of tree communities from the more easily measured seedling communities.

In our study, tree and seedling communities have the same variation patterns during succession. Our results suggest that the prediction abilities of functional traits from seedling to tree communities are dependent on the traits being measured and successional stage at the time of measurement. Most of the community level functional traits of trees can be well predicted by those of seedlings when all the successional stages are combined. Further studies are needed to explore the relationships between trees and seedlings in community level functional traits for forests of different types and successional stages.

## Supporting Information

S1 Fig Similarity index between tree and seedling community during sucession. 18, 18-year-old fallow; 30, 30-year-old fallow; 60, 60-year-old fallow; OG, old growth forest; All, all the four successional stages combine.
(DOCX)

S1 Table Description of the study sites. (DOCX)

S2 Table Description of the tree and seedling community in each successional stage. (DOCX)

S3 Table Multivariate stepwise regression analysis between functional traits and environmental factors. (DOCX)
