# Supplementary figures and images for: Relationships between Community Level Functional Traits of Trees and Seedlings during Secondary Succession in a Tropical Lowland Rainforest
Source: PLoS One. 2015 Jul 14;10(7):e0132849. doi: 10.1371/journal.pone.0132849 (PMC4501726; doi:10.1371/journal.pone.0132849)

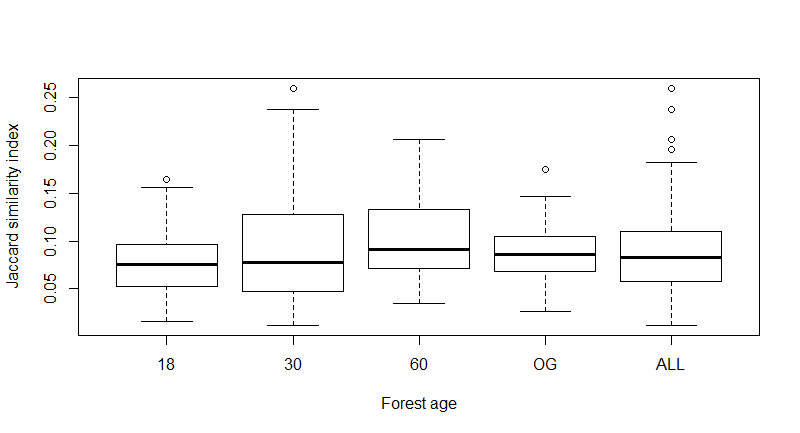
**S1 Fig. Similarity index between tree and seedling community during succession.**

Supplement: S1 Fig — 18, 18-year-old fallow; 30, 30-year-old fallow; 60, 60-year-old fallow; OG, old growth forest; All, all the four successional stages combine. (DOCX) [file pone.0132849.s001.docx]
