# Supplementary material for: Relationships between Community Level Functional Traits of Trees and Seedlings during Secondary Succession in a Tropical Lowland Rainforest
Source: PLoS One. 2015 Jul 14;10(7):e0132849. doi: 10.1371/journal.pone.0132849 (PMC4501726; doi:10.1371/journal.pone.0132849)
Supplement: S1 Table — (DOCX) [file pone.0132849.s002.docx]

**S1 Table.** **Description of the study sites.**

|  | **Used history** | **Cultivated species** | **Area of fallow** |
| --- | --- | --- | --- |
| 18-year-old fallow | shifting cultivation | *Manihot esculenta* Crantz | 7 ha |
| 30-year-old fallow | shifting cultivation | *Manihot esculenta* Crantz | 8 ha |
| 60-year-old fallow | shifting cultivation | *Manihot esculenta* Crantz | 7 ha |
| old-growth forest | None | None | 7ha |
