# Supplementary material for: Relationships between Community Level Functional Traits of Trees and Seedlings during Secondary Succession in a Tropical Lowland Rainforest
Source: PLoS One. 2015 Jul 14;10(7):e0132849. doi: 10.1371/journal.pone.0132849 (PMC4501726; doi:10.1371/journal.pone.0132849)
Supplement: S2 Table — (DOCX) [file pone.0132849.s003.docx]

**S2 Table. Description of the tree and seedling community in each successional stage.**

|  | | **18-year-old fallow** | **30-year-old fallow** | **60-year-old fallow** | **old-growth forest** |
| --- | --- | --- | --- | --- | --- |
| **Tree community** | **Dominant species** | *Aporusa dioica, Cratoxylum cochinchinense, Melastoma sanguineum, Engelhardia roxburghiana* | *Aporusa dioica, Melastoma sanguineum, Engelhardia roxburghiana, Cratoxylum cochinchinense* | *Psychotria rubra, Castanopsis carlesii, Symplocos poilanei, Engelhardia roxburghiana* | *Ardisia quinquegona, Mallotus hookerianus, Koilodepas hainanense, Vatica mangachapoi* |
|  | **Number of species(species per 400 m^2^)** | 34 ± 5 | 35 ± 7 | 42 ± 6 | 59 ± 8 |
|  | **Number of individuals (individuals per 400 m^2^)** | 454 ± 95 | 399 ± 167 | 355 ± 72 | 250 ± 50 |
|  | **Total DBH (cm per 400 m^2^)** | 1433.7 ± 276.9 | 1401.1 ± 483.2 | 1434.7 ± 277.1 | 1051.9 ± 150.5 |
|  | **Mean DBH (cm per 400 m^2^)** | 3.63 ± 0.54 | 4.21 ± 0.99 | 4.78 ± 0.67 | 6.60 ± 1.35 |
| **Seedling community** | **Dominant species** | *Psychotria rubra,*  *Aporusa dioica,*  *Helicteres angustifolia,*  *Canthium horridum* | *Aporusa dioica,*  *Psychotria rubra,*  *Helicteres angustifolia,*  *Breynia rostrata* | *Psychotria rubra,*  *Ardisia quinquegona,*  *Diospyros cathayensis,*  *Castanopsis carlesii* | *Vatica mangachapoi,*  *Cyclobalanopsis patelliformis,*  *Ardisia quinquegona,*  *Syzygium hancei* |
|  | **Number of species (species per 4 m^2^)** | 16 ± 5 | 15 ± 4 | 20 ± 6 | 18 ± 6 |
|  | **Number of individuals (individuals per 4 m^2^)** | 60 ± 26 | 50 ± 31 | 61 ± 29 | 42 ± 19 |
|  | **Total height (m per 4 m^2^)** | 2009.6 ± 986.1 | 1780.5 ± 1276.0 | 2425.1 ± 1585.6 | 1541.3 ± 1268.9 |
|  | **Mean height (m per 4 m^2^)** | 35.2 ± 146 | 37.0 ± 15.2 | 41.1 ± 22.9 | 36.1 ± 21.1 |
