# Supplementary material for: Relationships between Community Level Functional Traits of Trees and Seedlings during Secondary Succession in a Tropical Lowland Rainforest
Source: PLoS One. 2015 Jul 14;10(7):e0132849. doi: 10.1371/journal.pone.0132849 (PMC4501726; doi:10.1371/journal.pone.0132849)
Supplement: S3 Table — (DOCX) [file pone.0132849.s004.docx]

**S3 Table. Multivariate stepwise regression analysis between functional traits and environmental factors.**

| **Succession** | **Community functional traits** | **CO** | **SWC** | **TN** | **TP** | **AN** | **AP** | **pH** | **SOM** | **P** |
| --- | --- | --- | --- | --- | --- | --- | --- | --- | --- | --- |
| 18-year-old fallow | △ SLA |  |  | 0.09 |  |  |  |  |  | 0.45 |
|  | △ LDMC |  |  |  | - 0.03 |  |  |  |  | 0.13 |
|  | △ CC |  |  |  | -0.11 |  | -0.09 |  |  | 0.09 |
|  | △ LNC |  |  | 0.03 | -0.06 |  |  | 2.11 |  | 0.10 |
|  | △ LPC |  |  |  |  |  |  |  |  | 0.93 |
| 30-year-old fallow | △ SLA |  | 0.22 |  |  | -0.59 |  |  | 0.80 | 0.07 |
|  | △ LDMC |  |  | 0.02 |  | -0.10 | -0.04 |  |  | 0.33 |
|  | △ CC | -0.23 |  |  | 0.73 |  | 0.18 | -1.71 | -0.84 | 0.06 |
|  | △ LNC | 0.13 |  |  | 0.53 |  |  |  |  | 0.34 |
|  | △ LPC | -0.15 |  |  | 0.45 |  |  | -1.55 | -0.31 | 0.06 |
| 60-year-old fallow | △ SLA |  |  | -0.10 |  | 0.26 | -0.22 |  |  | 0.29 |
|  | △ LDMC |  |  | -0.03 |  |  |  |  |  | 0.08 |
|  | △ CC |  |  | 0.11 |  | 0.30 |  |  | -0.53 | 0.22 |
|  | △ LNC |  |  |  |  |  |  |  |  | 0.97 |
|  | △ LPC |  |  | -0.10 |  | 0.11 |  |  |  | 0.93 |
| Old growth forest | △ SLA |  |  |  |  |  |  | -3.74 | -0.98 | 0.08 |
|  | △ LDMC |  |  | -0.12 |  | 0.19 |  |  |  | 0.06 |
|  | △ CC |  |  | -0.17 |  |  |  |  |  | 0.07 |
|  | △ LNC | 0.28 |  |  |  | 0.28 |  |  | -0.76 | 0.06 |
|  | △ LPC |  |  |  |  | 0.15 |  |  | 0.22 | 0.07 |
| All stages | △ SLA | 0.25 | 0.20 | 0.07 | 0.26 |  |  |  |  | 0.06 |
|  | △ LDMC |  |  |  |  | 0.06 |  |  | -0.08 | 0.09 |
|  | △ CC | 0.19 |  |  |  |  |  |  |  | 0.07 |
|  | △ LNC | 0.17 |  | 0.07 |  | 0.15 |  |  | -0.29 | 0.06 |
|  | △ LPC | -0.06 |  | -0.04 |  | 0.07 |  |  |  | 0.10 |
